# Development of Genomic Microsatellite Markers in *Carthamus tinctorius* L. (Safflower) Using Next Generation Sequencing and Assessment of Their Cross-Species Transferability and Utility for Diversity Analysis

**DOI:** 10.1371/journal.pone.0135443

**Published:** 2015-08-19

**Authors:** Heena Ambreen, Shivendra Kumar, Murali Tottekkad Variath, Gopal Joshi, Sapinder Bali, Manu Agarwal, Amar Kumar, Arun Jagannath, Shailendra Goel

**Affiliations:** Department of Botany, University of Delhi, Delhi, 110007, India; National Institute of Plant Genome Research, INDIA

## Abstract

**Background:**

Safflower (*Carthamus tinctorius* L.), an Asteraceae member, yields high quality edible oil rich in unsaturated fatty acids and is resilient to dry conditions. The crop holds tremendous potential for improvement through concerted molecular breeding programs due to the availability of significant genetic and phenotypic diversity. Genomic resources that could facilitate such breeding programs remain largely underdeveloped in the crop. The present study was initiated to develop a large set of novel microsatellite markers for safflower using next generation sequencing.

**Principal Findings:**

Low throughput genome sequencing of safflower was performed using Illumina paired end technology providing ~3.5X coverage of the genome. Analysis of sequencing data allowed identification of 23,067 regions harboring perfect microsatellite loci. The safflower genome was found to be rich in dinucleotide repeats followed by tri-, tetra-, penta- and hexa-nucleotides. Primer pairs were designed for 5,716 novel microsatellite sequences with repeat length ≥ 20 bases and optimal flanking regions. A subset of 325 microsatellite loci was tested for amplification, of which 294 loci produced robust amplification. The validated primers were used for assessment of 23 safflower accessions belonging to diverse agro-climatic zones of the world leading to identification of 93 polymorphic primers (31.6%). The numbers of observed alleles at each locus ranged from two to four and mean polymorphism information content was found to be 0.3075. The polymorphic primers were tested for cross-species transferability on nine wild relatives of cultivated safflower. All primers except one showed amplification in at least two wild species while 25 primers amplified across all the nine species. The UPGMA dendrogram clustered *C*. *tinctorius* accessions and wild species separately into two major groups. The proposed progenitor species of safflower, *C*. *oxyacantha* and *C*. *palaestinus* were genetically closer to cultivated safflower and formed a distinct cluster. The cluster analysis also distinguished diploid and tetraploid wild species of safflower.

**Conclusion:**

Next generation sequencing of safflower genome generated a large set of microsatellite markers. The novel markers developed in this study will add to the existing repertoire of markers and can be used for diversity analysis, synteny studies, construction of linkage maps and marker-assisted selection.

## Introduction

A member of the family Asteraceae, Safflower (*Carthamus tinctorius* L.) is a diploid (2n = 24), mostly self-pollinating dicot with an estimated haploid genome size of 1.4 GB [1]. The crop is grown in wide geographical zones across the world [2] with Kazakhstan and India currently dominating safflower production [3]. It is a multi-purpose crop employed for diverse uses such as dye production, edible oil extraction and for medicinal applications [4]. It has also been exploited for production of biofuel and industrial oil [5,6]. Recently, transgenic safflower has been employed as a plant factory for production of important pharmaceuticals of human interest such as insulin and apo lipoprotein [7–9]. Considering the desirable oil composition of safflower and its resilience to dry conditions, it can serve as an important source of edible oil especially in arid regions of the world. However, undesirable features such as low yield, spiny nature and susceptibility to several biotic stresses have reduced its cultivation in several regions including India [10].

Conventional breeding programs in several crop species have resulted in the development of cultivars with improved yield and increased resistance to several diseases. Improvements can be achieved more efficiently and faster through analysis of global genetic diversity existing in the crop for selection of elite genotypes and by molecular breeding approaches [11]. Application of molecular markers in crop breeding has proven to be a powerful method for improvement of several crop species [12]. A prerequisite for successful implementation of molecular breeding in crops is the availability of strong molecular marker-trait association [11]. A comprehensive program to increase yield is essential for safflower improvement [13]. However, safflower genetics and genomics are largely unexplored and scarcity of reliable molecular markers in safflower is a major limitation for development of effective molecular breeding programs in the crop [14, 15].

A wide range of dominant markers such as random amplified polymorphic DNA (RAPD), amplified fragment length polymorphism (AFLP), inter-simple sequence repeat (ISSR) and sequence-related amplified polymorphism (SRAP) have been used for assessing the genetic diversity of safflower [16–20]. However, the dominant inheritance pattern of these markers does not allow detection of allelic information, which is important for crop breeding. Conversely, co-dominant markers allow detection of allelic diversity but in safflower, the repertoire of co-dominant markers is limited. Since their discovery in early 1980’s, microsatellite markers or SSRs (simple sequence repeats) have gained importance owing to their co-dominant inheritance, multi-allelic nature, wide genome coverage, high reproducibility, high polymorphic index, adaptability to automation, high throughput genotyping as well as efficient transfer to closely related species making them valuable tools for genetics and breeding [21–23]. In safflower, earlier studies used conventional methods of library enrichment or EST databases for development of microsatellite markers. Chapman et al. [24] generated 104 polymorphic EST-SSRs for linkage mapping in safflower. Naresh et al. [25] reported five EST-SSRs used for testing the purity of safflower hybrids. Hamdan et al. [26] isolated 64 polymorphic genomic SSRs from an enriched genomic library of safflower. However, these methods have high development cost and low throughput restricting the use of microsatellite markers [27]. Next generation sequencing (NGS) provide resources for high-throughput SSR development at a lower cost [28, 29]. Mining of NGS data for development of microsatellite markers has been exploited in a variety of plant species viz., pigeon pea, chrysanthemum, chokecherry, grass pea [30–33]. In safflower, Lee et al. [34] reported thirty polymorphic microsatellite markers derived from pyro-sequencing data while Pearl et al. [35] reported first set of 244 single nucleotide polymorphism (SNP) markers. Nonetheless, till date, only 203 polymorphic SSR markers have been reported indicating an urgent need for enrichment of robust co-dominant markers in safflower.

The genus *Carthamus* includes 18 species of which, *C*. *tinctorius* L. is the only cultivated species [1]. The wild species of *Carthamus* are known to harbor several agronomically desirable traits, which were lost during the course of safflower domestication [36–38]. Transferability of microsatellite markers to closely related species and genera would assist in the identification of marker-trait associations, which could be used for introgression of desirable loci from the wild species to cultivated safflower thus broadening its gene pool. Such markers would also be useful for synteny studies, identification of progenitor species and the study of genome evolution in the crop.

The current study exploited the efficiency of next generation sequencing data for analysis of microsatellite fraction present in safflower genome and derivation of a large set (5,716) of novel microsatellite markers for safflower. A subset of 325 microsatellite markers was experimentally validated using twenty-three geographically diverse safflower accessions. In addition, cross species transferability of polymorphic markers was assessed. Markers generated in this study would serve as important resources for population genetics, construction of linkage maps and marker-assisted selection in the crop.

## Materials and Methods

### Plant material and genomic DNA extraction

An accession of *C*. *tinctorius* L. (PI No: 560175) with high oil content (44%) was used for Illumina paired-end sequencing. A geographically diverse set of 23 safflower accessions belonging to seventeen countries was used to test the developed microsatellite markers. Cross species transferability of polymorphic microsatellite markers was tested using nine wild relatives of *C*. *tinctorius* L., including the probable progenitor species (*C*. *oxyacantha* and *C*. *palaestinus*). Seed samples were obtained from USDA-ARS, WRPIS, Pullman, WA, USA and IPK Gene Bank, Germany. Detailed information on plant material used in this study is given in Table 1.

**Table 1:** Details of plant material used in the study.

Species	PI number^#^	Origin	Ploidy level	Somatic chromosome number
**Variability study**				
*C tinctorius* L.	613514	Australia	2X	24
*C tinctorius* L.	401477	Bangladesh	2X	24
*C tinctorius* L.	305188	India	2X	24
*C tinctorius* L.	401583	India	2X	24
*C tinctorius* L.	544007	China	2X	24
*C tinctorius* L.	262447	Kazakhstan	2X	24
*C tinctorius* L.	304408 (a)	Pakistan	2X	24
*C tinctorius* L.	304408 (b)	Pakistan	2X	24
*C tinctorius* L.	388905	Iran	2X	24
*C tinctorius* L.	388907	Iran	2X	24
*C tinctorius* L.	306687	Israel	2X	24
*C tinctorius* L.	340096	Turkey	2X	24
*C tinctorius* L.	576991	Germany	2X	24
*C tinctorius* L.	613465	Spain	2X	24
*C tinctorius* L.	306599	Egypt	2X	24
*C tinctorius* L.	306596	Egypt	2X	24
*C tinctorius* L.	239041	Morocco	2X	24
*C tinctorius* L.	348915	Canada	2X	24
*C tinctorius* L.	537111	Mexico	2X	24
*C tinctorius* L.	560169	USA	2X	24
*C tinctorius* L.	560172	USA	2X	24
*C tinctorius* L.	560175	USA	2X	24
*C tinctorius L*. *var*.*inermis* Schweinf	CART 87	Romania	2X	24
**Cross-species amplification**				
*C*. *oxyacantha*	426184	Afghanistan	2X	24
*C*. *palaestinus*	235663	Israel	2X	24
*C*. *boissieri* Halacsy	Cart 85	Greece	2X	20
*C*. *tenuis subsp*. *Foliosus*	Cart 91	Cyprus	2X	20
*C*. *glaucus subsp*. *anatolicus*	Cart 43	Israel	2X	20
*C*. *lanatus*	235666	Portugal	4X	44
*C*. *lanatus subsp*. *Creticus*	CART 10	-	4X	44
*C*. *lanatus subsp*. *Lanatus*	CART7	-	4X	44
*C*. *lanatus subsp*. *turkestanicus*	426181	Afghanistan	4X	44

^**# Genotypes with CART ID are obtained from IPK while the rest have been procured from USDA.**^

Leaf material was harvested from 10-week-old plants of each accession and total genomic DNA was isolated using CTAB method [39]. The qualitative and quantitative analysis of extracted DNA was done by electrophoresis on a 0.8% agarose gel and using a NanoDrop spectrophotometer (NanoDrop, Wilmington, DE).

### Next generation sequencing using Illumina HiSeq^TM^ 2000 and de novo assembly

A 100bp-paired end sequencing run was performed on HiSeq 2000 platform (Illumina, USA) by Macrogen Inc. (Korea). The FastQ files containing the raw data were submitted to the sequence read archive (SRA) at National Centre for Biotechnology Information (NCBI) under the accession number SRP050023. High quality reads were identified from genomic sequencing data using NGS QC Toolkit at default parameters [40]. *De novo* assembly was performed using SOAPdenovo version 2.04 (http://soap.genomics.org.cn//soapdenovo.html) [41] at various K-mer values (21, 27, 33, 39, 45, 51, 57 and 63). The assembled contigs from each run were pooled and clustered using CD-HIT (http://weizhong-lab.ucsd.edu/cd-hit/) [42]. Contigs with >90% sequence similarities were considered redundant and removed.

### Identification of microsatellites, functional annotation and development of primer pairs

The assembled sequences were mined for perfect microsatellites using an in-house developed Perl script (S1 Script). The script contains separate modules for different SSR types (di- to hexa-nucleotides) and provides the details in terms of SSR type, repeat number, start and end position of repeat in the query sequence, total length of repeat and the complete sequence. Clustering was performed using CD-HIT on the identified sequences harboring microsatellites (clustering criteria; similarity > = 90% and 80% length coverage) to remove redundancy. Imperfect and compound SSR types were not included in the analysis. Functional annotation of the retrieved microsatellite sequences was performed using web-based automated annotation pipeline, FastAnnotator using default parameters (http://fastannotator.cgu.edu.tw/) [43].

Sequences containing microsatellites with repeat length of ≥ 20 bases (10 units for di-; 7 units for tri-, 5 units for tetra- nucleotides) and optimal flanking regions (≥ 30 bases on both flanks of microsatellite) were used to design primers. The web-based program, BatchPrimer3 version 1.0 (http://probes.pw.usda.gov/batchprimer3/) [44] was used for designing primer pairs with following parameters: primer length 18–28 bases; product size ranging from 100bp-500bp; optimum annealing temperature between 50°C to 65°C and GC content of 40% to 80% with an optimum value of 60%. Other parameters were used at default setting. Blast+ version 2.2.26 (ftp://ftp.ncbi.nlm.nih.gov/blast/executables/blast+/2.2.26/) was used to query the previously reported SSR markers [24–26, 34] against the marker set for which primers were designed in the current study.BLASTN hits with an E value less than 1x10^-13^ were considered significant.

### Validation of microsatellites

Primers were synthesized at Integrated DNA Technology, USA. Genomic DNA of two safflower accessions (PI: 560172 and 560175) was used as template for standardization of PCR conditions. The PCR was conducted in a total reaction volume of 15μl containing 50 ng of template DNA, 1X PCR buffer, 2mM of MgCl_2_, 0.2mM of each dNTP, 0.3mM each of forward and reverse primers and 1.25 U of Taq DNA polymerase (Biotools, Spain). Amplifications were performed in a Veriti thermal cycler (Applied Biosystems, USA) with the following cycling conditions: Initial denaturation at 96°C for 5 mins followed by 28–30 cycles of 96°C for 45s, primer annealing temperature (Tm; optimized for each primer pair; ranging between 55°C to 65°C) for 30s, DNA extension at 72°C for 1 min and a final extension at 72°C for 7 mins. The generated amplicons were analyzed on 2% agarose gel for product size and amplicon quality.

### Genotyping and cross species transferability

Primer pairs producing a clear unambiguous band were used for genotyping a panel of 23 safflower accessions (Table 1). The polymorphic markers were further assessed for their cross species transferability in nine wild relatives of *C*. *tinctorius* L. (Table 1). For polymorphic markers, M13 tailing of the PCR product was adopted as described earlier [45, 46]. The labeled PCR products were analyzed on 6.5% PAGE using 4300 DNA analyzer system (LICOR, USA).

### Marker diversity analysis

Statistical analyses of genetic data [Average number of alleles per locus (*Na*), gene diversity per locus (*He*), observed heterozygosity (*Ho*) and polymorphic information content (PIC)] of microsatellite markers were evaluated using POWERMARKER version 3.25 (http://www.powermarker.net) [47]. Cluster analysis for polymorphic microsatellite loci across the tested panel was performed using DARwin version 5.0.158 (http://darwin.cirad.fr/darwin) [48] based on simple matching coefficient.

## Results and Discussion

### Genome sequencing of *C*. *tinctorius* L.

**Fig 1:**
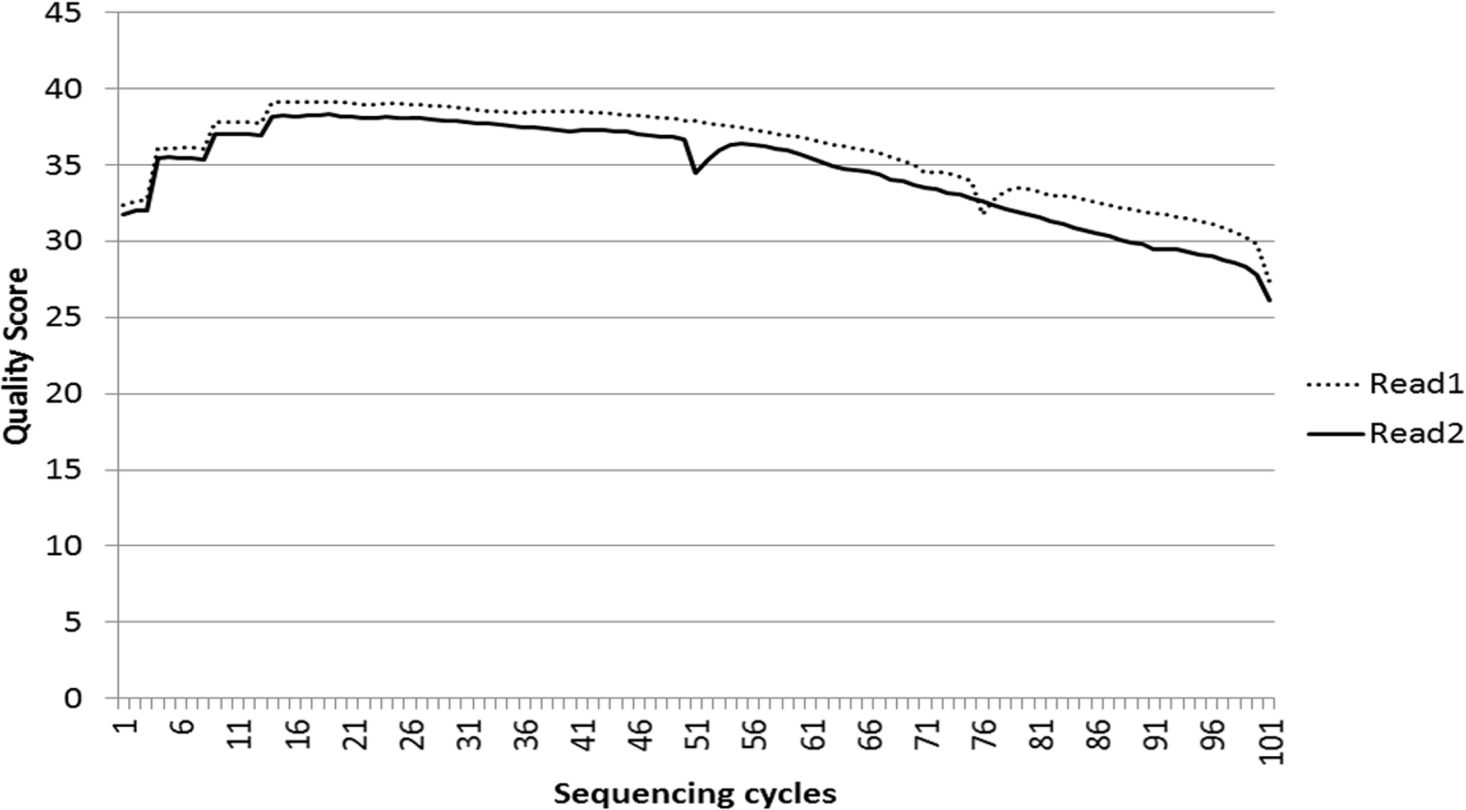
Quality score during sequencing cycles of HiSeq^TM^ 2000.

Illumina paired-end technology was used for sequencing the safflower genome. We obtained 48,502,680 raw reads with an average read length of 101 bases which provided ~3.5X coverage of the genome. The average quality score (Q) of raw reads was >25 in all the sequencing cycles (Fig 1). The raw sequences were checked for sequence artifacts such as low quality reads and adaptor contamination using NGS QC Toolkit [40]. A total of 44,164,564 (91.06%) high quality filtered reads were obtained with 98.1% bases showing a Q value of >20.

Assembly of the quality filtered and trimmed sequences were performed using SOAPdenovo version 2.04 [41]. Various k-mer values (21, 27, 33, 39, 45, 51, 57 and 63) were used for assembly and assembled sequences at each k-mer were pooled resulting in 4,078,739 contigs. Redundancy in contigs was removed using CD-HIT program [42], which resulted in 2,043,956 contigs with an average contig length of 264bp. Around 90% of the contigs were found in the size range of 100bp to 499bp. Length distribution of the obtained contigs is given in Table 2. Contigs <100 bases in length were excluded from further analyses. The generated safflower genome showed an average GC content of 38%, which is in consonance with several plant species such as Arabidopsis (36%), grape (34.6%), tomato (36.2%), potato (35.6%), rubber (36.2%) and mungbean (34.69%) [49–53].

**Table 2:** Length distribution of clustered sequences.

Read length (base pair)	Number
100–499	1843988
500–999	183848
1000–1999	15330
2000–2999	586
3000–3999	126
4000–4999	46
5000–5999	19
6000–6999	9
7000–7999	1
8000–8999	1
9000–9999	1
10000–10999	1
**Total**	2,043,956
**Average length (base pair)**	264
**Total nucleotides clustered**	540749137

### Discovery of microsatellites

An in-house developed Perl script (S1 Script) was used for mining perfect microsatellites from the clustered genomic data. Perfect repeats were selected as these are known to have higher mutation rates than imperfect loci and are expected to therefore, yield more polymorphism [54]. Additionally, more allelic variation is observed with increasing number of repeats [55]. Thus, sequences with repeat length < 20 bases were not analysed as these may not be significantly polymorphic. Following these criteria, we identified 31,390 microsatellite sequences which were further filtered to remove redundancy using CD-HIT and a non-redundant set of 23,067 putative microsatellite loci was obtained.

**Fig 2:**
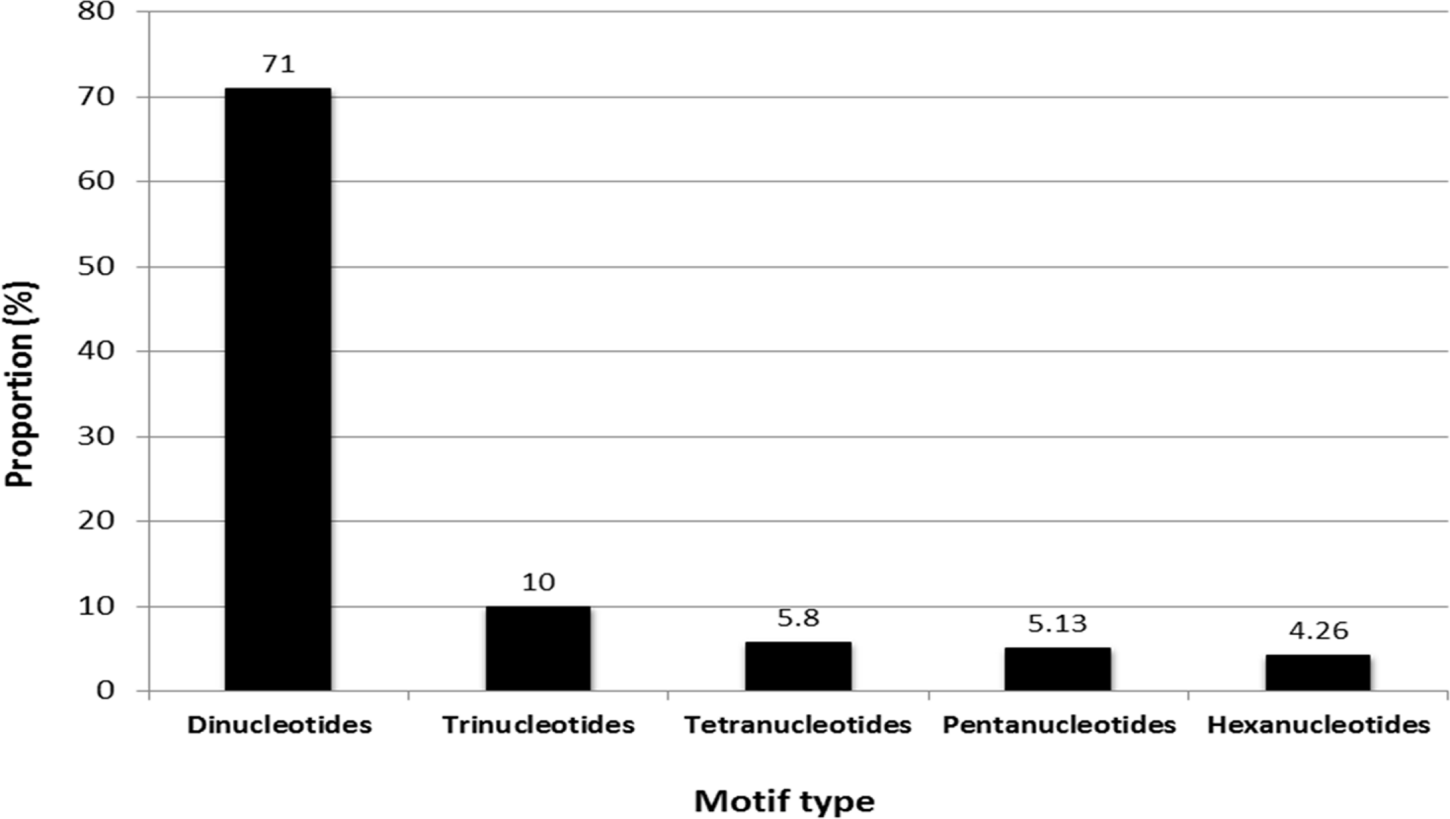
Distribution analysis of major classes of microsatellites in safflower genome.

**Fig 3:**
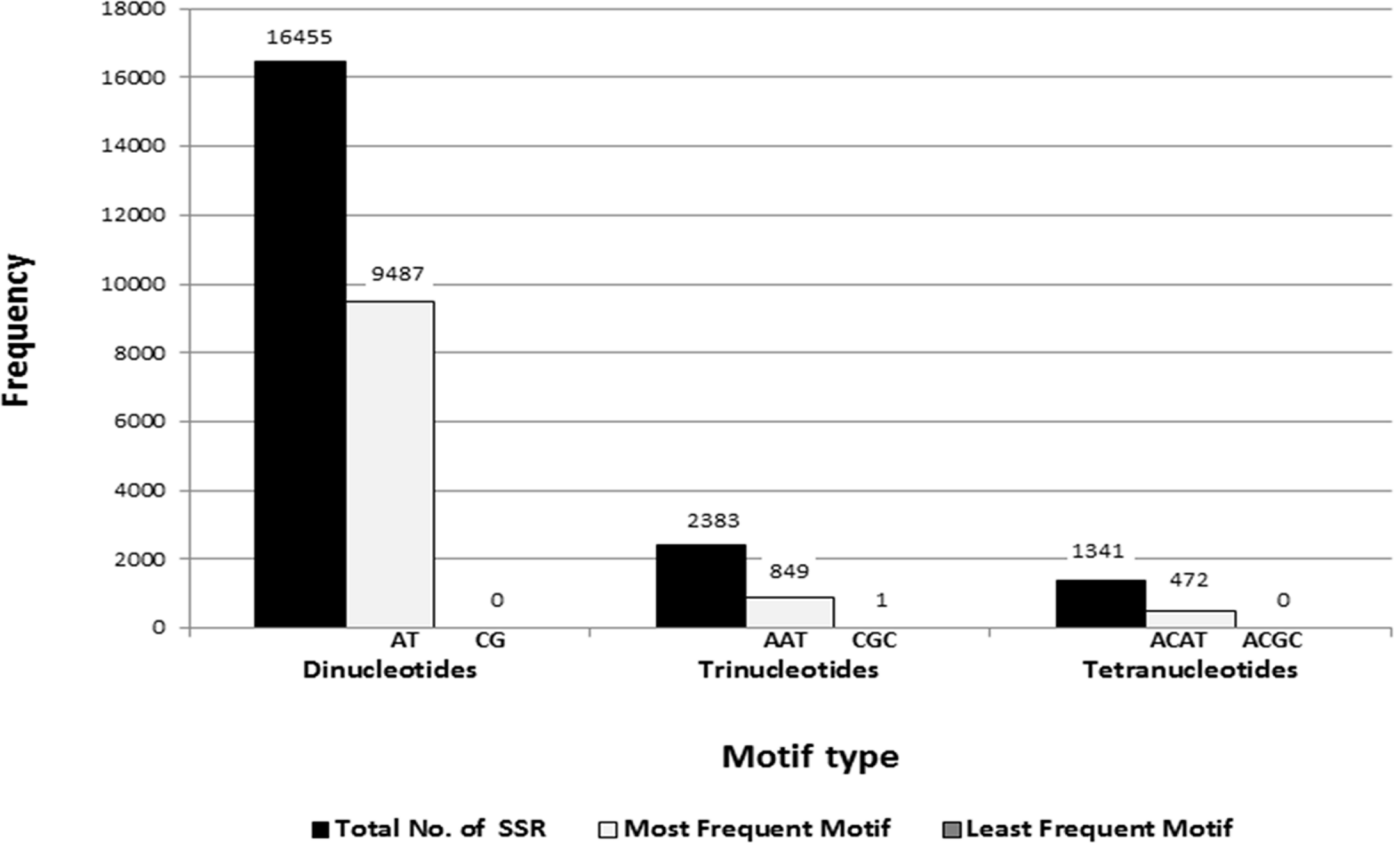
Frequency distribution of SSRs with most and least represented repeat motif in each class.

**Fig 4:**
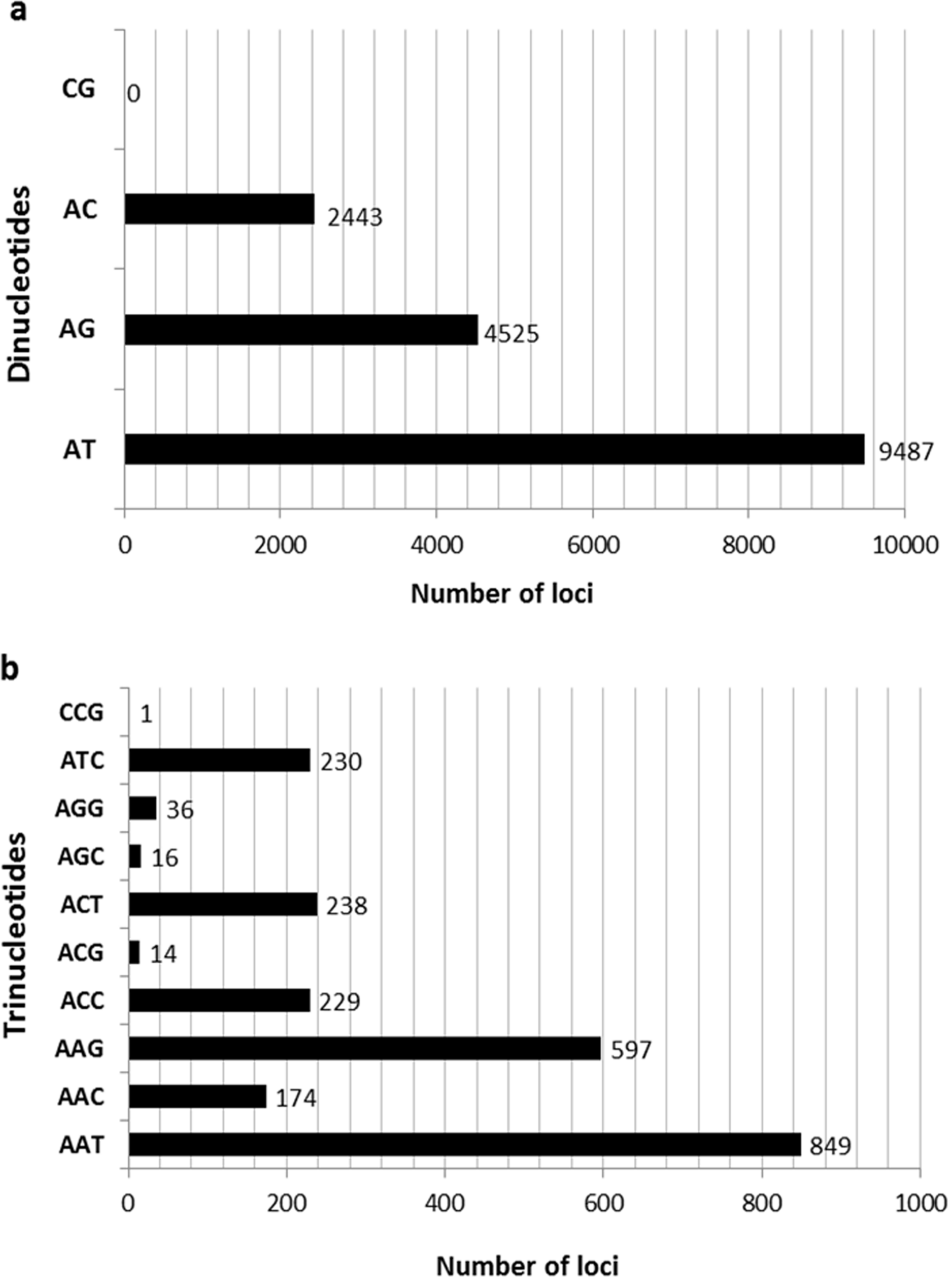
Characterization of di- and tri-nucleotide microsatellites discovered in safflower genome.

Significant heterogeneity was observed in frequency, motif type and repeat length of SSRs in safflower. Di-nucleotides were the most frequent type of repeats representing 71% of total SSRs, followed by tri- (10%), tetra- (5.8%), penta- (5.13%) and hexa-nucleotide repeats (4.26%; Fig 2). Di-nucleotides are known to be the most represented SSRs in genomes of several plant species viz., pigeonpea, mungbean, sweet potato and sesame [30,54,56,57]. The safflower genome was highly enriched with AT/TA repeats accounting for 57.65% of all the dinucleotide motifs followed by AG/TC (27.5%) and AC/GT (14.8%) repeats. This is in consonance with the observations of Lee et al., [34] who isolated microsatellites based on pyro-sequencing of the safflower genome. In general, plant genomes have been reported to be rich in AT repeats [58–60]. We did not obtain any CG/GC repeat in the analyzed data (Fig 3 and Fig 4A). Among tri-nucleotide repeats, AAT was the most common motif (35.6%) followed by AAG (25%). However, Lee et al., [34] reported ACC to be the most frequent tri-nucleotide repeat (27%) in safflower. This variation could be due to differences in the quantum of data generated in the two studies. While Lee et al. [34] reported 1100 contigs with SSRs, we obtained a significantly higher number of SSR-containing contigs (23,067) which may represent a more accurate distribution of SSR frequencies. Another factor could be the inherent bias observed in individual sequencing runs [61,62] that could have led to differences between the two studies. The least frequent tri-nucleotide motif was CGC for which only one locus was detected and it represented the only GC-rich trimer repeat obtained in the current study (Figs 3 and 4B). Low frequency of GC-rich repeats was also reported in genomic sequences of other crops [52, 53]. Among tetra-nucleotide repeats, the ACAT motif was the most predominant (Fig 3).

**Fig 5:**
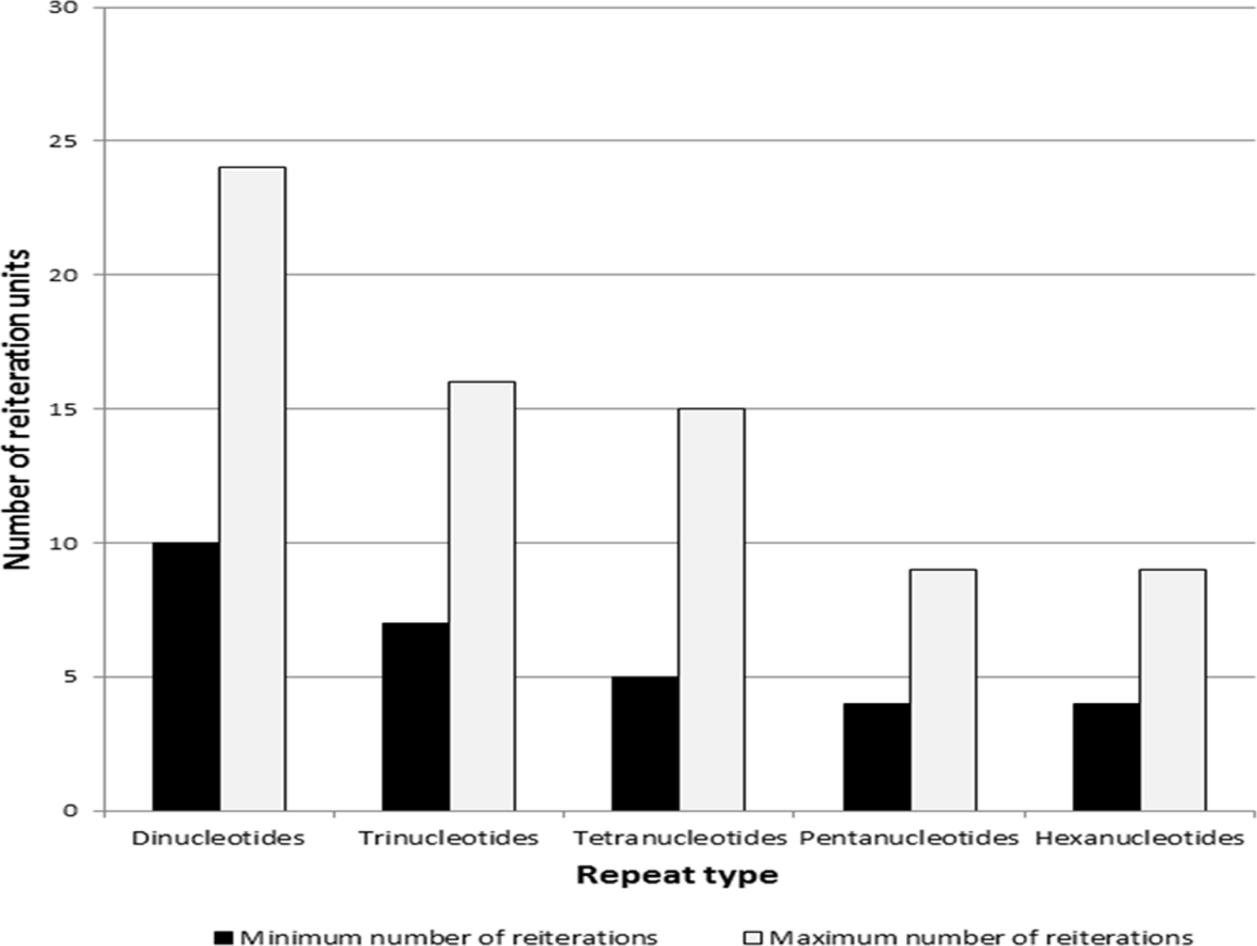
Reiteration units observed for various classes of SSRs in safflower genome.

The microsatellite motifs were also assessed for their repetitive unit length. The reiteration number of a SSR motif ranged from 4 to 24 and di-nucleotides were found to have greater number of reiteration units, which gradually decreased in higher motif types (Fig 5).

### Functional annotation of microsatellite sequences

**Fig 6:**
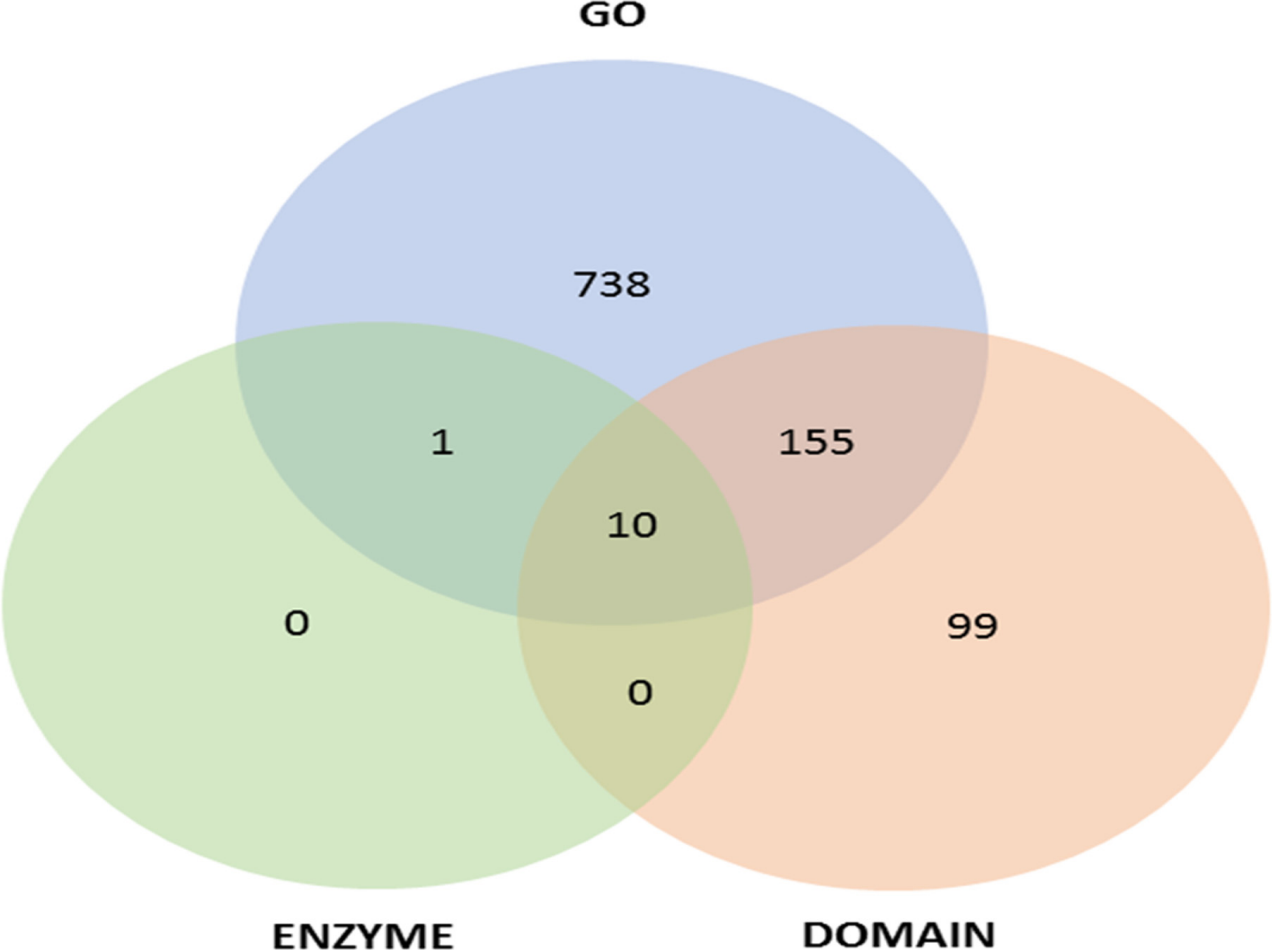
Functional annotation of microsatellite sequences.

**Fig 7:**
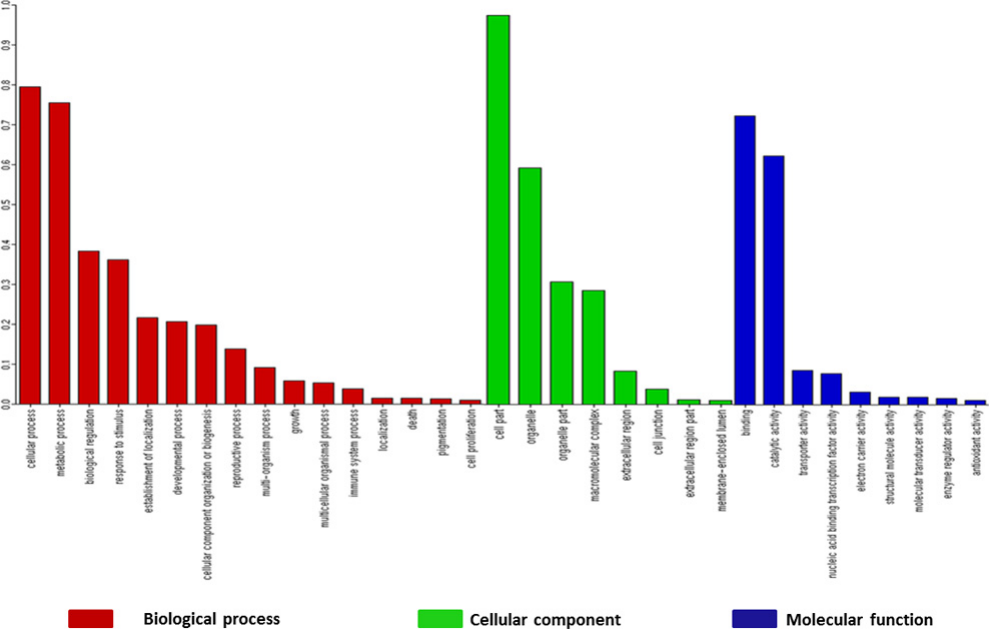
Distribution of annotated genomic microsatellites of *C*. *tinctorius* L. among the Gene Ontology functional classes: Biological process, Cellular component and Molecular function (Level 2).

In order to study the potential functional significance of 23,067 microsatellite sequences, annotation was performed using FastAnnotator [43], which reported the average length and GC content of these sequences to be 300 bp and 30%, respectively. More than 50% of sequences (~ 13,000) were greater than 200 bp in length and the N50 of these sequences was 383 nucleotides. Out of the total set analyzed, 2,611 sequences were found to have similarity with sequences in the NCBI non-redundant protein database and 1,003 sequences (4.3%) were found to have at least one functional annotation. Around 738 sequences were assigned gene ontology (GO) while 99 sequences contained at least one domain. Ten sequences were found to be common among all annotation categories while 155 sequences were found to be common among GO and domain categories. Only one sequence was found to share the GO and enzyme annotation (Fig 6). S1 Table provides detailed information regarding annotated SSR sequences. Nine hundred and four contigs were mapped to gene ontology terms with 767, 695 and 757 assignments distributed under biological process, cellular component and molecular function ontology, respectively. Fig 7 shows GO classification (Level 2) of annotated microsatellite sequences. In the biological process ontology class, cellular and metabolic processes were predominant. Under molecular function class, binding and catalytic activity were the most abundant while cell part and organelle have the highest number of assignments under cellular component class. Similar results for distribution of GO terms were obtained in earlier studies on safflower floral transcriptome [63]. The present study thus reports a novel set of microsatellites, which might be correlated with the expressed components of safflower genome.

### Validation of SSR markers

Primer pairs were designed for microsatellite loci using BatchPrimer3 version 1.0 [44] which allows detection of SSR motifs and designs primers from flanking regions. Out of 23,067 microsatellite loci identified above, 5,737 loci were recognized with sufficient flanking region and fulfilled the criteria for primer design (*see* Methods). Homology search of 5,737 microsatellite loci against the previously reported SSRs in safflower [24–26,34] was performed. The BLASTN results revealed that 14 and 7 sequences had significant similarity (E value < 1x10^-13^) with SSRs previously reported by Chapman et al. [24] and Lee et al. [34], respectively. These sequences were removed from the analysis leading to the identification of 5,716 novel microsatellites in safflower.

A subset of 325 microsatellite loci, designated as NGSaf_1 to NGSaf_325, was chosen for experimental validation and included di- (58), tri- (257) and tetra-nucleotide (10) repeats. These sequences have been submitted in the NCBI GenBank database under the accession numbers KM670560-KM670883 (S2 Table). High representation of trimeric repeats (79%) was selected to increase the probability of their presence in the coding regions [64]. It is believed that selective forces do not allow expansion of any repeat type other than trinucleotides in coding regions to avoid frame shift mutations that could alter protein functionality [60]. These repeats therefore, have a greater probability for stronger marker-gene/trait association and a high rate of transferability across species. Details of untested primer pairs are provided in S3 Table.

Out of 325 tested SSR primers, 294 (90.4%) generated high quality reproducible amplicons of expected size. Thirteen primer pairs failed to provide any PCR product and 18 primer pairs produced multiple amplicons, which were difficult to evaluate and were excluded from further analysis (S2 Table). The process of SSR development is subjected to attrition at each step. A mean 50% attrition between primer design and successful amplification of SSR loci has been reported in earlier studies [52, 65]. Lee et al. [34] tested 509 primer pairs in safflower, of which only 302 (59.3%) produced successful amplification. The higher rate of successful amplification in the current study (90.4%) could be due to improved generation and analysis of sequencing data.

### Polymorphism analysis and cross species transferability of SSR markers

Based on their geographical origin, 23 safflower genotypes from 17 countries of the world (Table 1) were selected for testing the discriminatory potential of validated microsatellite markers. Out of the 294 microsatellite loci which produced robust amplification, 93 (31.6%) were polymorphic among the studied genotypes (Table 3). The average number of alleles (*Na*) per locus varied from 2 to 4 while the mean observed heterozygosity (*Ho*) and mean gene diversity (*He*) were 0.0494 and 0.3746, respectively. The low level of observed heterozygosity might be attributed to the highly self-pollinating nature of the crop. Sixty-eight polymorphic markers revealed 2 alleles, 23 markers yielded 3 alleles while 2 markers detected 4 alleles among the 23 accessions. In total, 213 alleles could be identified in the assessed genotypes using 93 polymorphic SSR loci. Polymorphism information content (PIC) was calculated for each marker and ranged from 0.0416 (NGSaf_43, 91, 115) to 0.5602 (NGSaf_69) with a mean PIC of 0.3075 (Table 3).

**Table 3:** Characteristics of 93 polymorphic SSR markers developed for *Carthamus tinctorius* L.

Locus	Repeat motif	Forward primer sequence	Reverse primer sequence	Product size (bp)	Ta (°C)	Allele number (*Na*)	Gene diversity (*He*)	Heterozygosity (*Ho*)	PIC	GenBank accession number
NGSaf_2	(TC)8	ATCCTACGTGTCAACTTCCCAT	AATCAACAGCAACTAACGCTGA	251	62	2	0.2580	0.0434	0.2247	KM670561
NGSaf_9	(ACA)7	AGTCCAACATAGCTCGGATTTC	GACCACTCTCTTCTCCGTCATC	248	62	2	0.4848	0.0434	0.3673	KM670568
NGSaf_12	(CGG)7	TTCATCAACAAGTTACAAGGGC	ATTCCCTCTCCTACCCACCTAC	288	62	2	0.3402	0	0.2823	KM670571
NGSaf_13	(AGC)7	GGCACAAAGGGATGTTTCTT	TTCTATGATGGGAGGAACTGGT	108	58	2	0.0831	0	0.0797	KM670572
NGSaf_14	(CAT)7	ACCCATGTCAATGTACTCTTCGT	AATCTCCTCCATCTCCTCATCA	196	58	2	0.1219	0.0434	0.1144	KM670573
NGSaf_15	(TGT)7	CACTCCCTCGTGCAGCTT	ATTCCCAATGCTCCAACAATC	141	60	2	0.3147	0.0434	0.2652	KM670574
NGSaf_20	(CATT)5	AGCAAAGTGCTTGCTGACAA	AAGTTCAATTGAGCCCGATG	243	60	2	0.4990	0	0.3745	KM670579
NGSaf_22	(AG)9	CCCTCGAGTAAAACTCAAGTCA	AAATGGATTGGTGGGTTTCA	151	60	3	0.2344	0	0.2200	KM670581
NGSaf_23	(AC)9	ATATCCTCCTCCGCCGATAA	ATCAACAGGCGTCTCAACCT	169	60	2	0.3856	0	0.3112	KM670582
NGSaf_28	(ATC)7	TATGATCTTGTCCGGCCTCT	ATGGCGGATGTTCATAAAGG	177	62	2	0.0831	0	0.0797	KM670587
NGSaf_34	(CAG)7	CATCAAAATCATTGGTTGCTTG	TCTTTCACACACTTCTAAGGCAAA	165	60	3	0.5950	0	0.5261	KM670593
NGSaf_39	(GATG)5	AATGCTCCAGCTTTCGACAT	TTCGGCCTTCGCTATGTAGT	247	60	3	0.3553	0	0.3159	KM670598
NGSaf_43	(CT)9	AAAGCGGTGTTAGTGCTGTGTA	TTTATATGGAAGTGGAAAGGGG	205	60	2	0.0425	0.0434	0.0416	KM670602
NGSaf_44	(AGG)7	CACTGTTGTGACCCTTGG	CACACACTTCTAAGGCAAACT	110	62	2	0.4653	0	0.3570	KM670603
NGSaf_45	(GCT)6	ACGCCTCTTCTTCTTTCCTTCT	CATTCATGGGTTTAGGTGGC	317	62	2	0.2268	0	0.2011	KM670604
NGSaf_48	(CTT)6	ACCCTGGATGACTGAAAACCTA	ATCAACACTCAGGCCCATATTC	259	58	3	0.4990	0	0.4337	KM670607
NGSaf_49	(CAT)6	CTCCTTTTGCTTTCTTGATTGG	TCACTCTTGCTGATTTGCTTTG	231	60	3	0.5368	0	0.4322	KM670608
NGSaf_56	(GAT)6	GCTAAAACAAGTGTGGCATCAA	AAGATGAAACCTCCCTCAAATG	172	60	2	0.1587	0	0.1461	KM670615
NGSaf_63	(GAA)6	ATCCGTTCTTCTTCTAGCACCA	CAGGTGGCATGTATTTTGTTTG	356	60	2	0.3147	0.0434	0.2652	KM670622
NGSaf_65	(CTC)6	GAGTGAAAAGAGGGAAGGGTTT	GGGGTTTGGAAAGGGTATTTA	144	60	2	0.2580	0.0434	0.2247	KM670624
NGSaf_67	(AGG)6	CTGTTCCACAAGACAAAAGCAA	TCAAGTCCCAATCTCAACCTTC	277	58	2	0.3147	0.0434	0.2652	KM670626
NGSaf_69	(TAC)6	TCTCATCAACGATAAAGCAGAATC	TCAACTTCATCTTTTCACGATTTC	299	62	3	0.6353	0.0454	0.5602	KM670628
NGSaf_73	(GTT)6	CCAAGGTGACGTGTTGTTCTT	CATCCTTCGATCCACGATAACT	225	60	2	0.1587	0.0869	0.1461	KM670632
NGSaf_83	(AGA)6	GCCAAACCCTAACACAGAATCA	CGGTTGTGCCCTAGCTTTTA	400	62	2	0.4914	0	0.3707	KM670642
NGSaf_84	(TCA)6	CGCCATCTCTCTCCTCTTCTTA	GGTGTGGAATGGAATGATGATA	133	60	2	0.3147	0.1304	0.2652	KM670643
NGSaf_89	(TCT)6	CTCGTAGCTGAGTTTATCGGTG	TGATTGCAGAGAGACTTGTTGA	169	60	2	0.4536	0	0.3507	KM670648
NGSaf_91	(TAT)6	CCACTTCTAGTTCGGGTTTCTG	CTGCTGTCATTTCATAGGGTTG	346	58	2	0.0425	0.0434	0.0416	KM670650
NGSaf_92	(TAC)6	AGAGGAGTCGATCTTGTGAAGG	GAGAGGTGATACGAGAAGCCAT	107	58	2	0.2268	0	0.2011	KM670651
NGSaf_94	(TCC)6	CCATCGAAACTCTACAAAACCC	AGGACAAAAGAGGGAATGATGA	400	60	2	0.4054	0.0434	0.3232	KM670653
NGSaf_98	(TAC)6	ATTCTCTGCATGTGGCTTTTCT	CTTGGTTACGGAGGAAGATTTG	276	60	2	0.4834	0	0.3665	KM670657
NGSaf_101	(TAT)6	GATTCCGTGCATTCTACACAAA	GAGGAACGAACTAGGAAGGGTT	141	62	3	0.4158	0	0.3741	KM670660
NGSaf_105	(TGG)6	CTGTAATTCTGCAACTGAGACCC	GAAGCCATTTTCCACGATTTC	157	60	2	0.3856	0	0.3112	KM670664
NGSaf_111	(CAA)6	AATACCCCGCCATCATAGATTA	GAGCTATTCGACAACCAAATCC	201	62	2	0.4914	0	0.3707	KM670670
NGSaf_114	(AAT)6	AGACAAATGCAACCACATTCAC	TGTTTATGATCCTTTCAGCCG	377	60	2	0.1587	0	0.1461	KM670673
NGSaf_115	(CTG)6	CCATCCAATACTGCAAATCTCC	AGTACCAGCAAGCTCCACCTC	240	62	2	0.0425	0.0434	0.0416	KM670674
NGSaf_117	(GAA)6	TTCCATTGAGTCCCATGAAGAT	TCTCTGTTCCACGTAGGGCT	190	62	2	0.0831	0	0.0797	KM670676
NGSaf_130	(TGG)6	TGCGACTTGTGTTTCTTCTTCCC	AAAAGCCGTCCGGTGAAATTG	371	62	2	0.4234	0	0.3337	KM670689
NGSaf_138	(TGT)6	AACCTGTGTACCATCTGCTAATTG	ATGAGATCCGAAGTCCATTGTT	160	60	2	0.0831	0	0.0797	KM670697
NGSaf_142	(TGT)6	GATGTTAACCTGTGTACCATCTGC	CGTCTAATGAACACTCAATCCAAA	442	60	3	0.4473	0.0454	0.3655	KM670697
NGSaf_145	(GGA)6	GAGCATGAAACGGAGAATTAGG	TCAACAGTAGCAGATCCTTCCA	358	60	2	0.4990	0	0.3745	KM670703
NGSaf_148	(CAT)6	CCATGATTTTATGCTCATCGTG	AGCAATAGCAGGTGCAGTGATAG	366	62	2	0.4536	0	0.3507	KM670706
NGSaf_151	(GAT)6	AGTCTTGGCCTGTAACCACTTATC	CATGAAGAGACGTTTGAAGCAG	377	60	2	0.4914	0	0.3707	KM670709
NGSaf_152	(CAA)6	GTTGTTCGTTTGCCCCAATCTG	CAGAGCCACAAGATGCCGAATTA	100	62	3	0.4682	0.0952	0.4149	KM670710
NGSaf_154	(GAT)6	TGATATCAATGGTATGATTTTCCTTT	GGATGGCGAACAAGATTACAA	500	62	4	0.5826	0	0.4938	KM670712
NGSaf_155	(AAG)6	AAGTATTTGAACAAGTGTACCGGC	AGATGATGAAGGAAGGAGGTAATG	335	58	2	0.3367	0	0.2800	KM670713
NGSaf_156	(CAC)6	AACTGCTTCTAGGGTTTTCCTCTT	CCCCAAATTACCAACTTCCATAC	294	62	3	0.2637	0.0434	0.2385	KM670714
NGSaf_158	(TAA)6	ATGTTTGTCCCACTCGGTCTTC	TAAGTAGCTGAAGTGCAAGGTCGT	274	60	2	0.1219	0.0434	0.1144	KM670716
NGSaf_164	(TGT)6	CATCTCAGCCTTTCTGTCTCCT	AAAATCGGTTGTTTGGTTGC	313	60	2	0.2268	0	0.2011	KM670722
NGSaf_173	(AGC)6	GTGGTTCGAGTCTGTTTATTTCCA	CTGCACTTTTGAGTTGTGTGTGAT	351	60	2	0.4536	0	0.3507	KM670731
NGSaf_178	(ATC)6	TAAGAAAGGGCACCATTGAAGTAG	GATATGAGCAGAGGAGTTTGTGC	128	62	2	0.4763	0	0.3629	KM670736
NGSaf_181	(GTA)6	TATGGTGATCGAAGAAGAAGACAG	ACTGAGCAATGAAGAGTTCCAGA	299	58	3	0.3279	0.0434	0.2954	KM670739
NGSaf_201	(TAT)6	GTTATTGTTGTCCGTGCAAGTAGT	GCTTGGTTCCTAGTCGTAGTTCAT	309	58	2	0.4914	0.0869	0.3707	KM670739
NGSaf_204	(AAC)6	GCCATGCCCATATACAAACAGATA	AAGAAATGGTTCCACCGAGTCA	350	60	2	0.3856	0	0.3112	KM670762
NGSaf_210	(TCT)6	TGATAGTAGCTTATTCCCTCAGCC	AACGGTGGTAGGATAGTTGACG	199	60	2	0.4977	0	0.3738	KM670768
NGSaf_211	(GAT)6	GGTCTGCAAGAGTAAGTGGGAG	CCAAATCCCTGCTACAAAACAT	132	60	2	0.4958	0.0909	0.3729	KM670769
NGSaf_236	(ATC)6	ACCTTGAGGGGTATTTGGGTAA	GTTGGTAGGGTGATCTTCGGT	293	62	2	0.4921	0	0.3710	KM670794
NGSaf_237	(ATT)6	GACATTCCACTTATGCCGGTAG	TGGGACAATGACTCTGTTTGAG	250	62	2	0.4962	0.9130	0.3731	KM670795
NGSaf_238	(CAT)6	AACAGTGGGCCTGATATGTTTT	CGGCTAATCCAAACCCTAGAAT	343	62	2	0.4395	0.0434	0.3429	KM670796
NGSaf_239	(ATT)6	CAAAACTTCTCAACCGTGAAC	GTAACATACCGATTTTCTTGGC	133	62	2	0.2777	0.2380	0.2391	KM670797
NGSaf_242	(AAG)6	GGAGGAGAGATTGAAGGTGAAG	CCAACCATCGTCTGAACTTTT	129	62	2	0.1937	0.0434	0.1749	KM670800
NGSaf_245	(ATG)6	TGAACATGGTAAAAACCCATACA	TCTCAAAGGAATGGGAATGG	109	62	2	0.4938	0	0.3718	KM670803
NGSaf_248	(TCT)6	CTGCATTTCTTCCCCATTATTATC	GTGTTTAAGGTTTCATTGCCTTCT	173	62	2	0.0831	0	0.0797	KM670806
NGSaf_255	(TCA)6	AGGTGTTGGGAGGACACTAAAA	CCGCTCTTAGCTAAACTCTTGC	224	62	2	0.4848	0.0434	0.3673	KM670813
NGSaf_257	(ACA)6	ACAGCACCTGCCATCTTCATTA	AGGCATGAGCTTCGATTTTAGC	239	62	2	0.3856	0	0.3112	KM670815
NGSaf_259	(ATT)6	ATCGGGCATCCACTACTAGCTT	TGTGAAGTGAAAGATGAAATTCAAGA	236	62	2	0.4763	0	0.3629	KM670817
NGSaf_261	(TTC)6	CGAATCTACCAAGATCTACCAAATCC	GATATGGCGGAGAAACCGTAAA	102	60	2	0.4990	0.0869	0.3745	KM670819
NGSaf_262	(TAT)6	AAACCTGTACGGACACGTATCAA	CGAATTCCATCTCGATTTCTATATTTG	132	60	3	0.5207	0.0434	0.4059	KM670820
NGSaf_264	(TTA)6	GATGATGAATTTTGGTCGAACG	CGTTTAATTGACGATAATGCATGTG	152	62	3	0.5051	0.0454	0.4511	KM670822
NGSaf_265	(AAT)6	GGTAAAGACGAAGTTCACGATGG	TCTATCGCCGCCTTCTTCAC	155	62	2	0.2508	0.0588	0.2193	KM670823
NGSaf_266	(TGA)6	TGGGAGTAAATTGGTGTTGAAGC	AAACGAAAACGGGAGAGATGAA	211	62	3	0.1975	0.0434	0.1847	KM670824
NGSaf_273	(GAA)6	ATAGTAATTTGTAGCGAAAAGCGAAA	TGGATTCCAGGATTAACATATTCTT	150	62	2	0.4914	0	0.3707	KM670831
NGSaf_276	(AGA)6	CCCATTTCAACTAAATTTCAAGC	TCTTCTTTCTGGGTGGCTTC	110	62	2	0.0867	0	0.0830	KM670834
NGSaf_279	(AAG)6	AGCCTCGAGTTAAGCCATGA	TTCACGCGTTCTCTGTAGGA	154	60	2	0.3254	0.0454	0.2724	KM670837
NGSaf_281	(AG)22	CAGGGACATCAGTTTGAGGAG	TGTTGGTGCTAACAAAGAACC	108	62	3	0.6258	0	0.5509	KM670839
NGSaf_282	(AG)18	TACCCTCAATGGGAGTACCG	GGTCATGAGCTCGAATGAGG	185	58	3	0.405	0	0.3679	KM670840
NGSaf_286	(GA)16	GCAAAATGGGAACACATTCA	TCTCCAGTGTAGCGTGTGAAA	243	58	3	0.6124	0	0.5432	KM670844
NGSaf_289	(AT)16	TGCCATTTCATTAGTCACGA	ACTTGTTTTTACATCGAATCCTTT	139	60	3	0.6200	0.9565	0.5435	KM670847
NGSaf_292	(CT)16	CGTAACCACATAATACTTGGAGAA	TCCATATCCTTGAAAACCCATT	175	62	2	0.3856	0	0.3112	KM670850
NGSaf_294	(CA)15	AAGTAACAGATAAGATTTCAACAGCTC	CGATGGTATGAATAGTCGTCGT	155	62	3	0.3494	0.0714	0.3084	KM670852
NGSaf_295	(CT)15	GCCTCTGCCTCTCTCTCTATTTT	CCTGTCGGTAGATCGAAGAAG	131	62	2	0.4716	0	0.3604	KM670853
NGSaf_296	(TC)15	TCTCTGACGCTATGGGACAA	TGTGAGGATATGTAGAATCCCATT	158	62	2	0.4536	0	0.3507	KM670854
NGSaf_300	(CA)13	TGCCTGATTCTTCACCTTCTC	TTAGCTGAAGCACTTTTGCTCTT	100	62	3	0.5141	0	0.4608	KM670858
NGSaf_301	(CA)12	TCTTTTCAAGTGTTGCATGAGC	TGATGGTCTTCTCGCTACCA	249	60	2	0.4567	0	0.3524	KM670859
NGSaf_306	(CT)14	GTCCAAGACCCGAGATGAAG	AGATTGGATTGCCGATAAATG	152	60	2	0.1244	0	0.1167	KM670864
NGSaf_307	(CT)14	ACCCAAATCATGCTTCAACA	GGTGTGGATAATCAACCACTTTC	151	58	2	0.3046	0	0.2582	KM670865
NGSaf_308	(CT)13	TAACCCGCATTCTTGGTTTT	CCCTATCTGTGTGAGCCTGA	157	60	2	0.4990	0	0.3745	KM670866
NGSaf_309	(GA)18	TTGCAACCCTTCAACAAAAA	TCGAAGCCTTACCTTCCTCA	172	62	3	0.4390	0.0454	0.3746	KM670867
NGSaf_310	(GA)16	GTCGATGGGAGTTTGAGAGG	CGATTGTGAGAGGGGTAGGA	204	62	2	0.4958	0	0.3729	KM670868
NGSaf_313	(GT)13	GGGGTTGTTTTCCAAGAGTTT	CAGACAACTTTATCCAACATAGGAC	155	60	3	0.4297	0	0.3854	KM670871
NGSaf_314	(GT)12	TTTTCCGACTAAAACCCCTTT	AGGGTGTTACACGCAAACTTC	100	62	3	0.4917	0	0.4076	KM670872
NGSaf_322	(TC)15	AGCCGAAGAGCAAGCAAAT	GAGATGTTATGGCGGTGGTG	105	62	2	0.5	0	0.375	KM670880
NGSaf_323	(TC)13	AAAGACTGTATTTGGGCTACGG	TTGAGATGCAACATGAATTGAA	168	62	2	0.4501	0.6842	0.3488	KM670881
NGSaf_324	(TG)15	TGTTTTCCGGTTATTTGAGGTT	GAAGATGCTCTAACGGACCAA	156	60	4	0.6228	0.0434	0.5457	KM670882
**Mean**						2.29	0.3746	0.0494	0.3075	

In our data, some repeat motifs were found to be more polymorphic among the studied accessions than other repeat types. The repeat motif ‘AG’ exhibited highest polymorphism (57.1%) among all analyzed repeats followed by ‘AAT’ (46.4%). Detailed information regarding motif type and percent polymorphism is given in Table 4. It has been reported that different taxa exhibit different preferences for SSR types [59]. This information would help in selection of motif types and increase the probability of finding polymorphic markers in safflower.

**Table 4:** Repeat motif type and their rate of polymorphism observed in the present study.

Repeat Motif type	Total primer per motif	Polymorphic primers	Percentage polymorphism
**Dinucleotides**			
AT	9	1	11
AG	28	16	57
AC	21	7	33
**Trinucleotides**			
AAT	26	11	42
AAC	32	10	31
AAG	62	12	19
ACC	16	3	19
ACG	10	2	20
ACT	39	10	25.6
AGC	15	3	20
AGG	21	5	24
ATC	26	8	30.7
CCG	8	1	12

**Fig 8:**
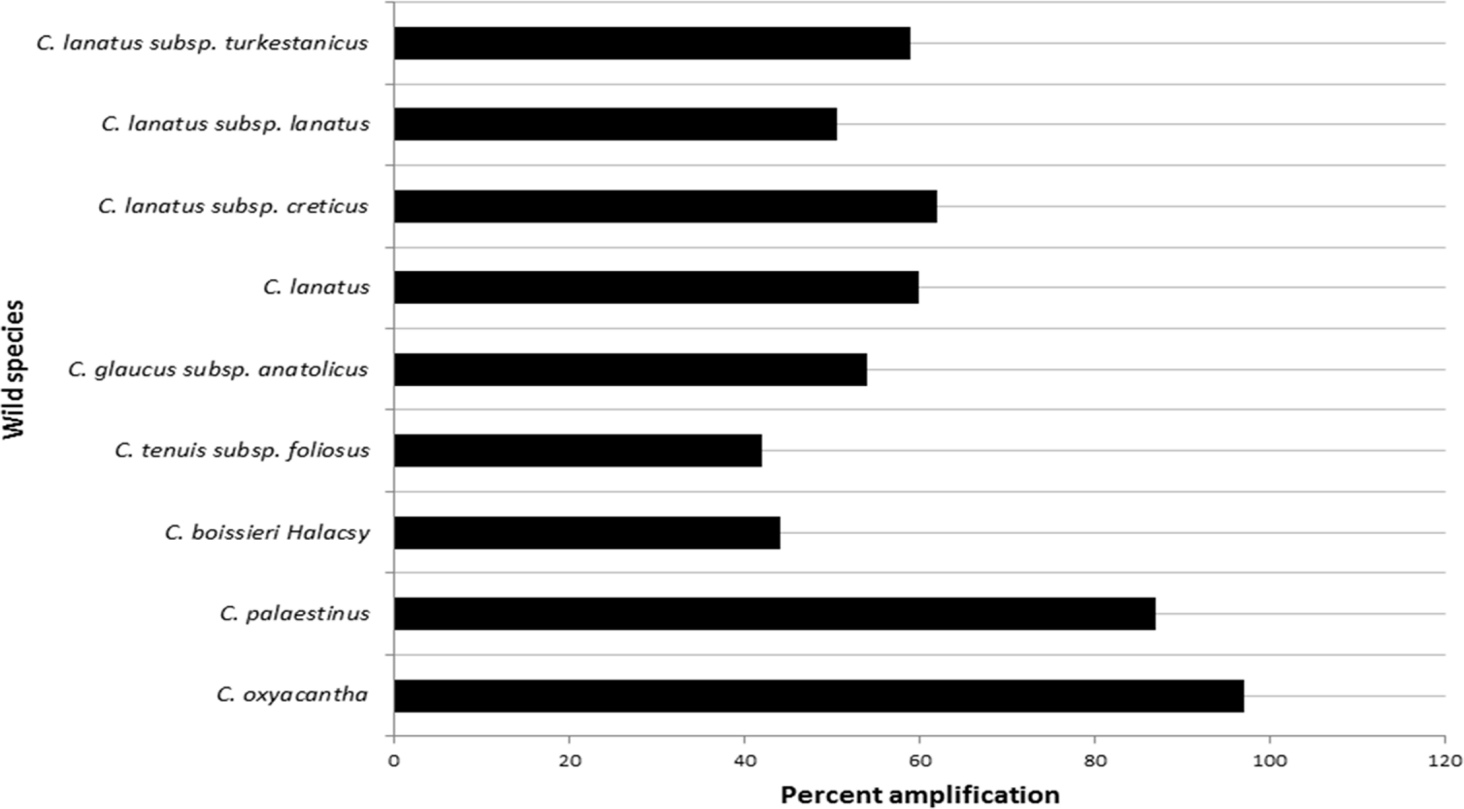
Cross-species transferability of 93 polymorphic safflower microsatellite markers in various species of genus *Carthamus*.

The cross species transferability of polymorphic SSR loci was also assessed in nine wild species of safflower (Table 1). Each primer pair, except NGSaf_307, was found to be amplifiable in two or more of the tested wild relatives (S4 Table). Twenty five SSR markers showed 100% transferability to all the wild relatives. The highest rate of cross transferability of markers was observed in *C*. *oxyacantha* (97%) followed by *C*. *palaestinus* (87%) while markers were found to be least transferable in *C*. *tenuis* (42%) (Fig 8). Based on cytogenetic studies, *C oxyacantha* and *C*. *palaestinus*, had been proposed as the possible progenitors of cultivated safflower [66]. The high rate of cross species amplification of SSR markers obtained in the present study supports the earlier observations on homology between these species and their possible contribution to the safflower genome [67].

### Cluster analysis for assessment of phylogenetic relationships in *Carthamus* sp.

**Fig 9:**
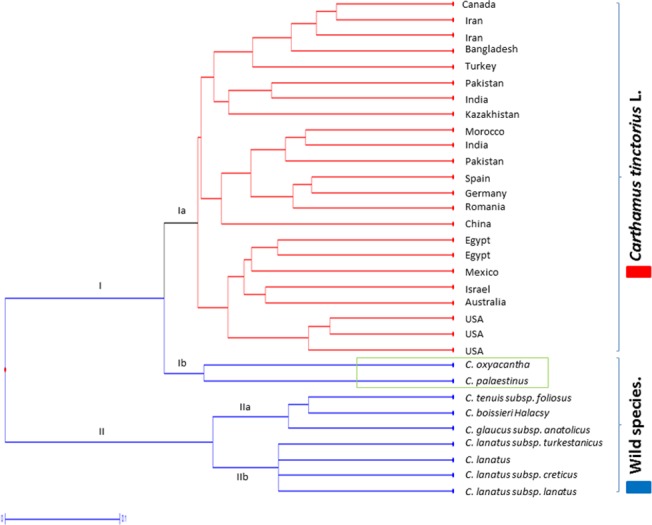
Phylogenetic dendrogram based on 93 polymorphic microsatellite markers, elucidating the genetic diversity and relationships among and between safflower accessions and its wild relatives.

Cluster analysis based on simple matching coefficient was used to assess the genetic relationships between *C*. *tinctoriu*s (safflower) genotypes and related wild species (Fig 9). The analysis grouped the studied accessions into two major clusters (I and II). All safflower genotypes, irrespective of their geographical origin, clustered in a single group (subgroup Ia) although some indicative groupings were observed for genotypes from USA and the European gene pool. Inclusion of more accessions from these geographical zones might be useful in identifying regional gene pools in the crop. The two wild species, *C*. *oxyacantha* and *C*. *palaestinus*, grouped together in subgroup Ib of cluster I. The clustering of *C*. *oxyacantha* and *C*. *palaestinus* along with *C*. *tinctorius* genotypes in cluster I supports the hypothesis that these wild species are more closely related to cultivated safflower than the other wild relatives. All the other wild species grouped together in Cluster II. Distinct clustering of accessions with differences in chromosome number was also observed. All *Carthamus* accessions with chromosome number n = x = 12 grouped together in cluster I. Cluster II segregated into two subgroups. Cluster IIa contained the diploid wild species with basic chromosome number = 10 (*C*. *glaucus* subspecies *anatolicus*, *C*. *boissieri* Halacsy and *C*. *tenuis*) while the tetraploid relatives (*C*. *lanatus* subspecies *creticus*, *C*. *lanatus*, *C*. *lanatus* subspecies *turkestanicus* and *C*. *lanatus* subspecies *lanatus*; basic chromosome number = 11) grouped in Cluster IIb.

Cross species amplification of microsatellite markers improves with decreasing phylogenetic distances [68]. The family Asteraceae is reported to have a low level of genetic conservation resulting in limited transferability of microsatellite markers across different genera [69]. Cross-genera transferability of microsatellite markers from sunflower has been shown to be inadequate and of limited use in safflower [14, 70]. Molecular markers developed in the current study demonstrated a high rate of interspecific amplification (ranging from 42% to 97%) within the genus *Carthamus*. We have also established the efficiency of these markers in elucidating the genetic relationships between members of the genus *Carthamus*. These markers could also be used for synteny studies between cultivated and wild species of safflower.

## Conclusion

In conclusion, our study provided an insight into the microsatellite components of safflower genome. Using next generation sequencing data, a large set of 5,716 novel microsatellite primers were designed of which, 325 markers were experimentally validated. Ninety-three markers were found to be polymorphic among the studied accessions. These markers were successfully used for genetic analysis in *C*. *tinctorius* L. and also showed significant cross species transferability in related wild species. Our data supports *C*. *oxyacantha* and *C*. *palaestinus* as the possible progenitors of cultivated safflower. We were also able to distinguish between various wild species with differing basic chromosome numbers. Markers generated in this study will enhance the current repository for safflower and would be useful in crop improvement programs. The current study also supports the efficiency of next generation sequencing data in providing faster and reliable resources for marker development in non-model crops.

## Supporting Information

S1 Script Perl_Scripts_SSRs. (ZIP)

S1 Table Annotation details of all SSR sequences (23,067). (XLSX)

S2 Table Details of 325 primers experimentally validated in present study. (XLSX)

S3 Table Details of 5, 391 SSR primers designed in present study. (XLSX)

S4 Table Locus specific details of cross-species transferability of polymorphic markers. (XLSX)
